# Efficacy and safety of long-term botulinum toxin treatment for acquired cervical dystonia: a 25-year follow-up

**DOI:** 10.1007/s00415-022-11343-0

**Published:** 2022-09-06

**Authors:** Martina Petracca, Maria Rita Lo Monaco, Tamara Ialongo, Enrico Di Stasio, Maria Luana Cerbarano, Loredana Maggi, Alessandro De Biase, Giulia Di Lazzaro, Paolo Calabresi, Anna Rita Bentivoglio

**Affiliations:** 1 Movement Disorders Unit, Fondazione Policlinico Universitario Agostino Gemelli IRCCS, 00168 Rome, Italy; 2 Medicine of the Ageing, Fondazione Policlinico Universitario “Agostino Gemelli”-IRCCS, Largo A Gemelli, 8, 00168 Rome, Italy; 3 Biochemistry and Clinical Biochemistry, Fondazione Policlinico Universitario Agostino Gemelli IRCCS, 00168 Rome, Italy; 4 Rehabilitation and Physical Medicine Unit, Fondazione Policlinico Universitario Agostino Gemelli IRCCS, 00168 Rome, Italy; 5 Department of Human Neurosciences, Sapienza University of Rome, Viale dell’Università, 30, 00185 Rome, Italy; 6 Department of Systems Medicine, University of Rome Tor Vergata, Rome, Italy; 7 Institute of Neurology, Università Cattolica del Sacro Cuore, Rome, Italy

**Keywords:** Botulinum toxin A, Acquired cervical dystonia, Long-term outcome, Safety, Efficacy

## Abstract

Botulinum toxin A (BoNT/A) is the first-line treatment for idiopathic cervical dystonia (ICD) and is widely used in the clinical setting. To date, scanty data are available on the effectiveness of BoNT in treating acquired cervical dystonia (ACD). Here we present a long-term follow-up of ACD patients treated with BoNT/A that focused on safety and efficacy. The study included subjects who had received at least six treatments of three commercially available BoNT/A drugs [abobotulinumtoxinA (A/Abo), incobotulinumtoxinA (A/Inco) and onabotulinumtoxinA (A/Ona)]. Safety and efficacy were assessed based on patients' self-reports regarding adverse effects (AE), duration of improvement of dystonia and/or pain relief. Global clinical improvement was measured on a six-point scale. 23 patients with ACD were administered 739 treatments (A/Abo in 235, A/Inco in 72, A/Ona in 432) with a mean number of treatments of 31 ± 20 (range 6–76) and duration of 10 ± 6 weeks (range 2–25). The mean dose was 737 ± 292 U for A/Abo, 138 ± 108 U for A/Inco and 158 ± 80 U for A/Ona. The average benefit duration was 89 ± 26 (A/Abo), 88 ± 30 days (A/Inco), and 99 ± 55 days (A/Ona) (*p* = 0.011); global clinical improvement for all sessions was 4 ± 1. ANOVA one-way analysis indicated that A/Ona had the best profile in terms of duration (*p* < 0.05), whereas A/Abo had the best pain relief effect (*p* = 0.002). Side effects were reported in 9% of treatments (67/739), with ten treatments (1%) complicated by two side effects. Most side effects were rated mild to moderate; severe side effects occurred following three treatments with the three different BoNT; two required medical intervention. No allergic reactions were reported. Even after 25 years of repeated treatments, all serotypes of BoNT demonstrate positive effects in treating ACD with long-lasting efficacy and safety.

## Introduction

Cervical dystonia (CD) is the most frequent form of dystonia. It is characterized by sustained, directional, involuntary movements of the head and neck that cause abnormal posturing along with head tremor and/or pain. Most cases of CD are defined as primary or idiopathic (ICD) [1]. Acquired dystonia (ACD) can be secondary to drug exposure, toxic, vascular, neoplastic, cerebral palsy (CP), brain injuries, post-infection/inflammation, functional, according to the etiological axis of the latest consensus on dystonia classification [2].

Depending on the prevailing direction of dystonic movement, CD can present in several forms, the most common of which are rotational torticollis and laterocollis, followed by retrocollis and anterocollis. The col-cap concept was adopted to assess CD in a more recent approach. It defined 11 dystonic subtypes of neck muscle contraction patterns and included lateral and sagittal shift among the abnormal postures [3]. CD is a long-term disease with rare (3–15%) spontaneous complete remissions, which usually occur during the first 3–5 years after first onset symptoms [4]. As CD is a long-term disease, it requires long treatment [5].

Botulinum toxin (BoNT) injections are the first-choice treatment for ICD; they are safe and effective overtime [6–9] and are also widely used in ACD.

Other treatment options are pharmacological or surgical; the latter include functional neurosurgery and selective peripheral denervation [10]. Anticholinergics, muscle relaxants and benzodiazepines are the most widely used oral drugs. Muscle relaxant drugs have been reported to be more effective in dystonia secondary to juvenile CP than in ICD, as well as clozapine use in tardive dystonia [11].

The BoNT benefit is maximal in simple patterned CD, such as rotational torticollis or laterocollis; however, complex patterns are more challenging and have a higher rate of treatment failure. Clinical presentation of ACD overlaps ICD; however, as the underlying pathophysiology is heterogeneous, the results of studies on ICD need to be confirmed in ACD [12], because few data have been reported on the use of BoNT in this group. A benefit in dystonia secondary to CP and Dopamine Receptor Blocking Agent (DRBA) exposure has been reported. However, the level of evidence is poor and long-term outcomes are reported in only one study on seven patients with a maximum follow-up of 5 years [13] and in a recent survey conducted on both idiopathic and acquired forms [14].

Here we present retrospective data on BoNT injection safety in a cohort of 23 patients affected by ACD with up to 25 years of follow-up.

## Methods

### Patient selection

We included 23 patients with ACD who frequented the Botulinum Toxin and Movement Disorders Outpatient Unit of the Fondazione Policlinico Universitario A. Gemelli IRCCS and collected clinical assessment and data about their response to the BoNT/A treatment.

Inclusion criteria were: (1) a diagnosis of ACD dystonia; (2) at least six consecutive treatments with one of the commercially available types of BoNT/A injections, i.e., onabotulinumtoxinA (A/Ona, Botox®, Allergan Inc, Irvine, CA, USA); abobotulinumtoxinA (A/Abo, Dysport®; Ipsen, Slough, Berkshire, UK); incobotulinumtoxinA (A/Inco, Xeomin ®; Merz Pharma, Frankfurt, Germany); (3) age ≥ 18 years.

Exclusion criteria were: (1) diagnosis of idiopathic or genetically proven CD; (2) inability to retrieve data; (3) previous surgical treatment for dystonia; and (4) failure to sign the informed consent form.

### Clinical assessment

In all patients, ACD diagnosis was confirmed by the coordinator of the Movement Disorders Unit. The diagnosis of ACD followed the etiological axis [2]: drug-induced; associated with Parkinson’s Disease (PD), other parkinsonism or other movement disorders; post-traumatic; metabolic; associated with CP, brain tumors or other neurological conditions.

Patients were classified according to their clinical CD presentation (torticollis, laterocollis, retrocollis, anterocollis or combined forms, dystonic tremor, shoulder elevation) and whether they had focal, multifocal, or generalized dystonia or hemidystonia. The procedures were in line with the ethical standards of the Helsinki Declaration (1964, amended most recently in 2008) of the World Medical Association.

### Treatment

This study evaluated the long-term use of flexible dosing regimens of botulinum toxin in a setting close to real-life clinical practice where the dose used is determined based on clinical needs. So, our Patients with ACD received injections of botulinum toxin A using flexible intervals (3–6 months) and dosing based on their needs and not in a fixed pattern in terms of time-lapse and units injected. The treatment was adjusted according to the therapeutic results and AE at each visit. BoNT/A was reconstituted in sterile saline solution sodium chloride as follows: (1) 100 U A/Inco in 2 cc (A/IncoBoNT concentration of 5 U per 0.1 cc); (2) 100 U A/Ona in 2 cc (A/OnaBoNT concentration of 5 U per 0.1 cc; (3) 500U A/Abo in 2.5 cc (A/AboBoNT concentration of 20 U per 0.1 cc). As in ICD, the starting doses were chosen according to the severity of dystonia, keeping the first dose as low as possible to minimize the risk of side effects.

In line with standardized procedures, a neurologist who was highly trained in movement disorders and BoNT use performed most of the treatments with instrumental targeting such as electromyography (EMG) guidance to increase the accuracy of the BoNT injections [15] and to detect activity and target posterior and deep neck muscles, with an expected amelioration of outcome [16]. The following muscles were injected based on the prominent dystonic pattern: trapezius, sternocleidomastoid (SCM), scalene, splenius capitis and levator scapulae.

We collected the following data: (1) treatment parameters (number of BoNT/A treatments; type of BoNT/A toxin; total dose and single muscle doses; EMG or US-guided targeting); (2) efficacy of the treatment in terms of latency, total duration, and duration of peak effect at each follow-up visit as reported by patients and/or next of kin. Latency was defined as the interval between injection and the first sign of improvement noticed by the patient. The total duration of improvement was defined as the interval between the first day of improvement and the last day of reported benefit; the peak effect duration was the number of days the patients experienced the best clinical effect. (3) In keeping with our previous studies [8, 17], therapeutic response as a global clinical assessment (GCA) was based on patient’s and/or of next of kin’s perception of improvement expressed as 0–100% compared with the baseline condition. CGA was rated from 0 to 6 (0 = no effect, 1 < 20%, 2 = 20–40%, 3 = 40–60%, 4 = 60–80%, 5 = 80–90%, 6 = complete resolution).

(4) Safety: as part of our routine activity, patients and their next of kin are encouraged to take note and report at the subsequent treatment session any side effects in terms of type, duration, severity and medical intervention, if required. The severity of side effects was quantified with a score ranging from 1 to 3 (1 = mild, 2 = moderate, 3 = severe), based on the patient’s subjective experience and the medical attention required. Side effects were considered ‘severe’ when they required medical intervention (i.e., severe dysphagia, neck muscle weakness with drooping head). Side effect duration was measured using a score ranging from 1 to 3 (1 = less than one week, 2 = between one week and one month, 3 = more than one month).

### Statistical analysis

Statistical analysis was performed using the Statistical Package for Social Science (SPSS), release 15.0. Continuous variables were expressed as the number of observations, mean ± standard deviation (SD), minimum and maximum value and categorical variables displayed as frequencies. We performed a one-way ANOVA analysis to compare the mean value of the efficacy parameters (i.e., latency, total duration of improvement, duration of maximum benefit, CGA) of the different types of BoNT/A. Statistical significance was considered for a *p* value < 0.05, and the corresponding effect size *η*^2^ was calculated.

## Results

We included 23 patients (56% were females). Dystonia was associated with the following conditions: chronic drug exposure (9 patients, pts), parkinsonism (4 pts), CP (6 pts), CNS traumatic injury (1 pt), post-infectious (1 pt), brain tumor (1 pt) and Tourette’s Syndrome (1 pt). At the first evaluation, the mean disease duration was 16.6 ± 17.5 years (range 1–47).

We administered 739 treatments: A/Ona 432, A/Abo 235, A/Inco 72. The mean number of injections was 32 ± 19 (range 9–76) and the mean BoNT/A treatment duration was 10.8 ± 6.3 years (range 3–25). During the observation period, dystonia progressively improved to resolution in one patient; nine patients withdrew from the treatment for different reasons, i.e., unsatisfactory outcome (3 pts), worsening of the neurological disorders causing dystonia (3 pts), difficulty in reaching the center (2 pts), death (1 pt). Two patients were lost during the follow-up.

Regarding the phenomenology of CD, rotational torticollis and laterocollis were the most frequent presentations, accounting, respectively, for 78 and 77% of all treatments, followed by shoulder elevation (37%), anterocollis (25%), retrocollis (24%) and dystonic tremor (9%). The most frequent complex pattern was rotational torticollis plus laterocollis plus shoulder elevation (23%).

Regarding body distribution, 52% of the patients presented focal CD, 22% segmental, 4% multifocal and 22% generalized dystonia. The most frequent association was with upper limb dystonia and cranial dystonia (respectively in 43% of the cases), followed by axial and dysphonic dystonia (respectively in 22% of the cases) and lower limb dystonia (in 13% of the cases).

Most treatments (73.5%) were performed without instrumental guidance. Injections were given under EMG guidance in 22% of the cases, under US guidance in 4% of the cases and double guidance was adopted in 0.5% of the cases.

### Efficacy

First, we conducted a global analysis by considering the total number of treatments performed. The mean BoNT/A dose-injected per session was: (1) A/Inco 111.6 ± 53.8 U (range 20–200); (2) A/Ona 160.9 ± 79.7 U (range 37.5–400), (3) A/Abo 725.6 ± 292.0 U (range 100–1400).

The mean doses for single muscles are detailed in Table 1. Mean CGA was 3.7 ± 0.9 (range 0–6), with a median value of 4. A CGA ≥ 3 was observed in 96% of the injections, with values between 4 and 6 in 78% of the treatments. CGA was totally ineffective in 2% of the injections.

**Table 1:** BoNT/A doses for each target muscle: mean values for the different type of BoNT; in bracket ranges are represented; (ranges are indicated in brackets)

	Target cervical muscles				
	SCM	Splenium	Trapezius	Levator scapulae	Scalenum
A/Inco	19.3 (10–30)	29.7 (10–55)	34.1 (10–80)	20.3 (10–30)	26.9 (7.5–75)
A/Ona	30.4 (10–80)	45.5 (10–150)	48.3 (10–150)	38.9 (15–70)	16.9 (10–50)
A/Abo	96.3 (40–250)	162.8 (40–500)	188.6 (40–500)	153.5 (40–240)	136.1 (40–200)

The mean latency was 6.2 ± 3.6 days (range 1–30), the mean overall duration of clinical improvement was 93.2 ± 29.2 days (range 0–180), and the mean peak effect duration was 76.4 ± 28.5 days (range 0–150).

A one-way ANOVA compared the mean values of the latency, the total improvement duration, the peak effect duration and CGA among the different types of BoNT/A. In this analysis, A/Ona showed a significantly shorter latency and more prolonged benefit period than the other BoNTs, with a small effect size (Table 2).

**Table 2:** Mean ± SD of efficacy, A/Inco, A/Ona, A/Abo

	A/Inco (*n* = 72)	A/Ona (*n* = 432)	A/Abo (*n* = 235)	*p*	Effects size (*η*^2^)
Latency (days)	6.1 ± 2.4	5.9 ± 4.1^a^	7.0 ± 3.0^a^	0.001	0.02–small
Maximum duration (days)	61.5 ± 25.7^a,b^	80.0 ± 29.0^a^	74.6 ± 26.7^b^	< 0.001	0.04–small
Total duration (days)	80.7 ± 25.6^a,b^	96.4 ± 30.5^a^	91.1 ± 26.7^b^	< 0.001	0.03–small
CGA score	3.6 ± 1.0	3.7 ± 0.9	3.7 ± 0.9	0.470	< 0.01–very small

CGA, clinical global assessment

Apex letters (^a,b^) locate the intergroups significant differences

### Long-term assessment

**Fig. 1:**
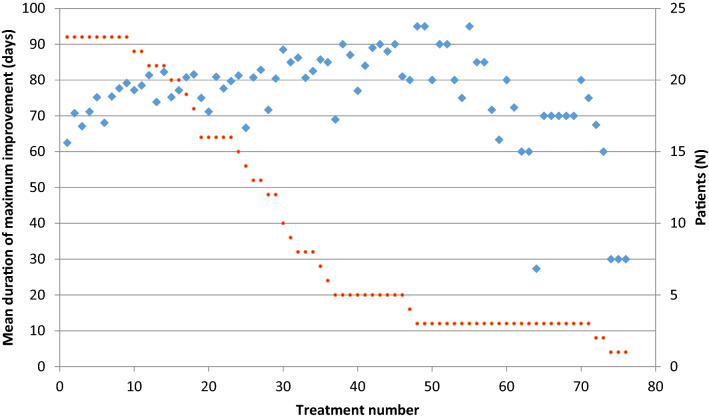
Mean duration of maximum clinical improvement overtime. In sky-blue: maximum benefit duration; in red: number of patients

Injection cycles were aggregated in terms of the number of treatments needed to assess long-term efficacy. The maximum clinical improvement mean duration, i.e., 63 ± 28 days at the first treatment, progressively increased overtime up to approximately the 21st treatment (mean 81 ± 30 days) and remained stable after that (Fig. 1).

The mean duration of the total improvement was 80 ± 29 days at after the first treatment. It progressively increased to 94 days after the 10th treatment, with subsequent progressive increase overtime to approximately the 30th treatment (mean 106 ± 31 days); it remained stable after that. The mean latency and the mean CGA did not change substantially overtime.

### Side effects

Side effects were reported in 9% of treatments (67/739), with ten treatments (1%) complicated by the occurrence of two side effects (Table 3). There were no allergic reactions. Most side effects were rated mild to moderate, severe side effects occurred in three treatments with the three different BoNT; two of them required medical intervention. In particular, the same patient experienced severe side effects twice after a long interval both with A/Abo and A/Ona; they consisted of severe posterior neck muscle weakness and dysphagia for less than a week. To deal with this condition, the patient required cervical collar support and adapted food. Another patient who was treated with A/Inco experienced severe cervical rigidity and pain at the injection site. The condition resolved spontaneously after a month. The side effects lasted less than a week in 40 cases, less than a month in 22 patients and more than a month in 5 cases. The frequency and severity of side effects did not change substantially overtime.

**Table 3:** Frequency of side effects expressed as % (number of cases) for the three BoNT/A

Side effects	Total	A/Inco	A/Ona	A/Abo
Total	9% (67)	0.8% (6)	4.7% (35)	3.5% (26)
Weakness of posterior neck muscles	7% (52)	0% (5)	3.7% (28)	2.5% (19)
Dysphagia	1.2% (9)	0	0.4% (3)	0.8% (6)
Pain at injection site	1% (8)	0.1% (1)	0.7% (5)	0.2% (2)
Rigidity	0.7% (5)	0.1% (1)	0.5% (4)	0
General weakness	0.1% (1)	0	0	0.1% (1)
Others	0.5% (4)	0	0.1% (1)	0.4% (3)

## Discussion

This study provides long-term safety and efficacy data regarding the administration of BoNT/A drugs to ACD patients with a very long follow-up (up to 25 years). Patients included in this study received up to 76 consecutive treatments with BoNT/A, with an average of 39 treatments per patient. This is comparable to the series reported by Vivancos-Matellano (17 years) [18] and Jochim (27 years) [14], which, however, included mostly idiopathic forms of CD. In this series, most treatments with BoNT/A had long-lasting therapeutic benefits; in fact, 96% of the patients reported an improvement of at least 40–60% (CGA ≥ 3), which was reportedly good in 78%. This result is very relevant as it overlaps the outcome of ICD [19, 20] in the long term.

The mean duration of clinical improvement was over 3 months (93.2 ± 29.2 days), and the peak effect was 76.4 ± 28.5 days, with an even longer duration when compared to our data on ICD, and consistent with the findings of other long-term studies (44). These efficacy parameters increased progressively with repeated injections: peak effect duration (from 63 days at (after) the first treatment to 81 at (after) the 21st treatment; total duration (80 days after the first treatment to 106 after the 30th). This trend is in line with the results of other studies (25), (32), (43), confirming the longer benefit duration following repeated BoNT/A injections. However, a recent retrospective study of a large cohort of patients with both idiopathic and ACD found stable efficacy over the years, with an increase in BoNT dosages only in the first year of treatment and subsequent stabilization (11). In conclusion, through repeated treatments the BoNT/A total dose and benefit duration increased, with stabilization after the 20th treatment cycle.

Overall, 22% of the injections were EMG-guided, mostly in CD complex patterns or in cases of previous treatment failure.

Side effects occurred following 9% of treatments (*n* = 67). They were mild and transient in most cases. Serious side effects occurred in 3 out of 739 treatments. The most frequent adverse effect in the present study was posterior neck muscle weakness, followed by dysphagia, pain at the injection site and rigidity. Neck muscle weakness and dysphagia were the most frequently expected side effects [21]: they were confirmed in case series that included ACD [14, 22]. In a study conducted on a sample of patients treated with A/Ona for parkinsonism induced and non-parkinsonism related CD, no difference in the prevalence of dysphagia was detected between the two groups [23]. In the longitudinal analysis, there was no evidence of an increase in either frequency or severity of side effects, thus confirming the hypothesis that the occurrence of a side effect does not cumulatively increase the likelihood that the patient will suffer from additional side effects in the future [24, 25]. Our data indicate that BoNT/A has an excellent long-term safety profile. These data are very relevant as they confirm those reported in patients with ICD [14, 21, 26].

We compared the results obtained with the three different BoNT/A available in our country. Due to its more recent introduction, fewer treatments were performed with A/Inco (i.e., 72 treatments versus 235 with A/Abo and 432 with A/Ona). Regarding the dosages per individual muscle, although there are defined recommendations for A/Ona and A/Abo in ICD [27], only one long-term study with A/Abo addressed this issue in ACD; it reported generally lower single muscle doses compared with our data [26]. Our patients received a mean dose per session of (1) A/Inco 112 U, (2) A/Ona 161 U and (3) A/Abo 726 U. This is consistent with most clinical studies as well as with the manufacturer's indications [22]. The mean dose of 161 U of A/Ona was lower than the doses used in previous long-term studies [21, 28], but slightly different from the doses used in the prospective cohort of the CD PROBE study conducted in 1000 US patients (mean dose 189 U) [29]. By contrast, the mean dose of 726 U of A/Abo was higher than the 500 U reported in a meta-analysis of 1202 patients [30] and in long-term studies [18, 26]. However, some studies report similar mean dosages of 800 U [22] or higher [31] in long-term treatment. The average dose of A/Inco seems to be lower than the dosages reported in only one long-term study in CD, in which the mean doses ranged from 151.4 U at the first injection to 192.2 U at the fifth infiltration [25]. The mean values of the efficacy parameters (latency, CGA, duration of maximum efficacy, time of total effectiveness) were compared among the three BoNT/A in a one-way ANOVA, with A/Ona showing a significantly shorter latency and a more extended period of a benefit than the other BoNTs. This result is only partially in line with the literature, as a longer-lasting effect has been attributed to the use of A/Abo due to the relative greater potency of the drug at the same doses (41). However, when corrected for η size, these differences were statistically very small or negligible. In support of this, in a recent systematic review of the Cochrane database no conclusive opinion emerged about the differences between BoNTs/A regarding the duration of the clinical effect (42).

Finally, our study provides information on the frequency of ACD in a tertiary Movement Disorder BoNT Clinic. Most of the cases in our series were drug-induced, followed by juvenile CP. These data are consistent with those of other patient series (22), including the Hannover Medical School database [32].

Also, the demographic and clinical characteristics of our sample overlapped those of ICD [1]: preponderance of female gender, mean disease duration, prevalent focal expression and association with cranial dystonia in cases of 'spreading' [33, 34]. Unlike what occurs in ICD, we observed spreading towards the upper limbs instead of the trunk [1]. To date, in tardive dystonia the cranio-cervical phenotype is the most frequent and its phenomenology does not seem to differ from that of idiopathic dystonia [35, 36]. Although it was previously assumed that in tardive CD, retrocollis and spasmodic head movements would be more frequently found [37], other case series including ours, showed a higher prevalence of torticollis and laterocollis with a primarily focal distribution [35, 38]. At our center, we chose a progressive approach in terms of dosage, i.e., we administered lower doses of BoNT/A in the first treatment cycles to limit potential side effects. This resulted in a progressive increase in drug doses overtime, as already described in our long-term series of ICD [39]. As already known, the treatment duration is influenced by the increase in dosages [40]. In drug-induced ACD (DRBA or levodopa), amelioration of CD progression overtime could be partly related to other intercurrent factors, such as discontinuation or reduction of therapies that putatively induced the dystonic symptoms (i.e., first or second-generation antipsychotics withdrawal or dopamine replacement therapy adjustments).

## Limits and conclusions

The main limitations of the present work are related to the retrospective design and the analysis of a non-homogenous sample for both etiologies and severity of ACD. Acknowledging the different characteristics of the different types of ACD and the small sample size of the patients included, we cannot draw definitive conclusions about phenotypes and treatment outcomes associated with the various conditions that cause CD. These factors can constitute confounding variables for data analysis and understanding. However, a 'real-life' study allowed us to longitudinally follow the clinical practice of BoNT infiltrations in ACD for a time window of up to 25 years, with an average follow-up of 8 years. This allowed us to understand the real impact of these treatments and the consistency of the safety profile overtime.

In conclusion, considering that the results of drug therapy aimed at treating CD are disappointing [10], this 25-year study provides information that is still lacking in the literature regarding the treatment of ACD and allows for an overview of outcome response, dose titration and expected side effects of the different subtypes of BoNT/A in a long frame. The results of our study contribute to data that demonstrate the safety of the drug in a wide range of conditions and overlap those regarding ICD in terms of both efficacy and safety, indicating that BoNT/A is the treatment of choice also for ACD.
